# Optimisation of the Extrusion Process through a Response Surface Methodology for Improvement of the Physical Properties and Nutritional Components of Whole Black-Grained Wheat Flour

**DOI:** 10.3390/foods10020437

**Published:** 2021-02-17

**Authors:** Yuxiu Liu, Miaomiao Liu, Shuhua Huang, Zhengmao Zhang

**Affiliations:** 1 College of Agronomy, Northwest A&F University, Yangling, Shaanxi 712100, China; yxliu@nwsuaf.edu.cn; 2 College of Food Science and Engineering, Northwest A&F University, Yangling, Shaanxi 712100, China; liumm2018@126.com; 3 College of Horticulture, Northwest A&F University, Yangling, Shaanxi 712100, China; hsh813@126.com

**Keywords:** black-grained wheat, whole-grain foods, extrusion, physical properties, nutritional components

## Abstract

Chronic undernourishment affects billions of people. The development of whole-grain food with high nutritional quality may provide a valuable solution to nutritional security. Black-grained wheat (BGW), as a rich source of protein and micronutrients, is a good raw material for value-added products. The objectives of this study were to investigate the effects of barrel temperature, feed moisture content, and feed rate on the physical properties and nutritional components of whole BGW flour extrudates and to optimise their processing conditions by using the response surface methodology. The increasing barrel temperature, feed moisture content, and feed rate affected the specific volume, expansion ratio, hardness, fracturability, water absorption index (WAI), water solubility index (WSI), and total starch content of the extrudates, but did not significantly affect the content of protein, ash, iron (Fe), zinc (Zn), copper (Cu), and manganese (Mn). The extruded wheat flour had a significantly higher content of Fe and Cu, and a lower total starch content than the unextruded flour under extrusion conditions. A significantly higher content of protein, ash, Zn, Cu, and Mn, and a significantly lower total starch content were found in the extruded and unextruded flours made of whole BGW than in those made of whole white-grained wheat. According to the significance of the regression coefficients of the quadratic polynomial model, the optimum extrusion parameters were as follows: a barrel temperature of 145.63 °C, feed moisture content of 19.56%, and feed rate of 40.64 g·min^−1^ in terms of the maximum specific volume, expansion ratio, fracturability, WAI and WSI, and the minimum hardness. These results may be used by food manufacturers to successfully develop extruded products from whole BGW flour, meeting consumer demands and needs.

## 1. Introduction

The nutritional requirements for humans mainly depend on plant-based food, such as that made from the edible portion of staple crops [1]. Wheat is one of the most important staple crops and makes a significant contribution to global food security. The global production volume of wheat in the 2019/2020 season amounted to more than 765 million tons, and approximately two thirds of this volume was consumed by humans [2]. The mineral content in grains is relatively low, which was attributed to its neglect because of the greater attention paid to high-yielding and high-resistance wheat cultivars [3,4,5]. Around 800 million people globally are still chronically undernourished, particularly in countries with a relatively high consumption of wheat derivatives; these people have weaker immune systems and may be at a greater risk of certain severe illnesses caused by viruses [6,7]. Thus, the interest in nutritious and functional foods made from wheat has increased among consumers [8]. Increasing the nutritional content of staple crops continues to be a huge task for worldwide scientists. Although some achievements have been made by biofortification in the recent past, in most cases, the expected goals have not been reached [1]. To meet the challenge of the nutritional crisis, nutritious properties of a raw food material should be used to develop food products with improved quality and health-related beneficial properties. Compared with traditional wheat, colour-grained wheat is characterized by significant levels of anthocyanins and essential nutrients (total phenolic acid content, selenium) [8,9,10,11,12,13,14,15,16,17,18]. Therefore, the exploitation and utilisation of colour-grained wheat products with health benefits not only would provide food with high nutritional quality but also contribute to the nutritional security of the ever-growing population.

Colour-grained wheat is a type of new germplasm resource in cereal crops, and its grain is of different colours, such as green, blue, purple, and black, as compared with common wheat (white or red) [8,9]. The Zn and Fe content of colour-grained wheat are about 108.54–142.68% and 8.57–42.96% higher than common wheat, respectively [19]. Among these, black-grained wheat (BGW) (ZP Black1 [9], Chinese black-grained wheat [20,21,22], six black wheat varieties including Hedongwumai [23], and Zhongpu Black 1 [24]) has a higher protein, total amino acids, essential amino acid, and micronutrient content, but lower total starch content, than white-grained wheat (WGW) (Wenmai 2504 [9], commercial US cultivars (Klasic and Yecora Rojo) [20,21], Dongjian white-grained wheat [22], 148 white wheat varieties including Yumai 58 [23], and PBW621 [25]), which is at a prime position for development as a nutritious and functional food that provides health benefits to humans. Several BGW cultivars (*Triticum aestivom* L.) have been released over the past 20 years, which are easy-to-grow and available crops [26]. The glycaemic and inflammatory profile of type 2 diabetes mellitus patients was improved by a daily substitution of BGW for a partial staple food [27]. The consumption of foods rich in anthocyanins has been related to lower risks of various diseases associated with oxidative stress [13]. In addition, food manufacturers would like to use new materials and new methods to make new nutritional products, in response to consumers’ demand for healthy, palatable, and nutritious foods. Therefore, the commercial prospects of the BGW product chain (grain-flour products) are bright because of the abundance and utilisability of the raw materials, its confirmed health benefits, the increasing consumer demand for foods that enhance their health, and the growing interest of both consumers and producers. BGW, as a new raw food material for value-added products [26], has not yet been used well in the food industry. This low use can be attributed to the limited research data available on its processing properties and product quality compared with data available on other types of grains, such as barley and oats. Therefore, it is necessary to study the processing technology that will yield the maximum value of the final nutrient content to meet the nutritional quality demand of the product and promote the development of BGW industrialisation.

The nutritional components of colour-grained wheat are close to those of its coloured seed coat. Most of the micronutrients exist in the peripheral part of the wheat grain [28]. The micronutrients decrease during wheat debranning [29]. Whole-wheat flour has a higher content of fibre, minerals, and micronutrients than refined wheat flour [30]. Whole-grain functional foods made with colour-grained wheat are promising new products [13]. In terms of its high nutritional quality and the competitive nature of the functional food market, an extruded whole BGW flour product has great potential to appeal to today’s consumers. Extrusion is an approach to process starchy materials for the production of snack foods [16]. Extrusion cooking provides an opportunity to change the proximate composition, such as the protein, moisture, and carbohydrates, of the raw materials and further affects the physical, chemical, and textural properties of the extrudates [31,32,33,34]. The characteristics of the extrudates depend on the physicochemical changes that occur during extrusion processing because of the effects of extrusion variables, such as the barrel temperature, screw speed, extrusion die temperature, feed moisture content, and feed rate [33,34,35,36,37,38,39]. Extrusion conditions with a high moisture content, low residence time, and low temperature improve the nutritional quality, whereas high extrusion temperatures (≥200 °C) and a low moisture content (<15%) deteriorate the nutritional quality [40]. Extrusion has been proposed as an effective method for the removal of antinutrients [32]. The extrusion of whole-wheat flour leads to an increase in the free sulfhydryl groups, while the total cysteine content remains almost unchanged [41]. Protein digestibility and free amino nitrogen have been improved by thermo-mechanical extrusion [42]. A significant decline in tannins with minimum oil loss was observed in flaxseed meal by using extrusion processing [43]. Extrusion significantly improves the water absorption index (WAI), water solubility index (WSI), and degree of gelatinization of corn and potato starch [38]. The nutritional quality is enhanced in cooked noodles made of extruded buckwheat flour (with 18–30% of the feeding moisture extruded) [39]. Phenolic acids (except for gallic acid) and flavonoids (especially luteolin) of Jizi439 black wheat bran were increased by extrusion (the third heating block temperature 110 °C, 25% feed water content, 140 rpm screw speed), and ultrasound [18]. The carotenoids content of the extruded products was decreased significantly, compared to the content in unextruded flour [16]. Extrusion conditions lead to starch melting, depolymerisation, and protein denaturation [44]. The temperature range of 115–146 °C can be used to design extruded products having sufficient soluble starch without massive insolubilisation of the proteins [44]. Starch gelatinisation and degradation were improved by the combination of a high temperature and shear stresses during extrusion of starchy materials [45]. 

The response surface methodology (RSM) consists of a number of statistical and mathematical techniques for process optimisation and improvement [46]. In this method, one or more dependent variables are used to check the effect of some independent ones for optimising the process. A mathematical model is developed by using RSM, which can describe the entire process with a restricted number of observations [37].

The effects of extrusion processing on the nutritional values of extrudates made from wheat flour, wheat–legume, and wheat–fish ingredients have been well documented [47,48,49]. To the best of our knowledge, however, little information is available on the nutritional quality of products made from BGW flour as affected by the extrusion processing variables. There is a possibility that extruding whole BGW flour not only improves its physicochemical and functional characteristics but also provides an alternative product to the existing healthy food products. The aims of this study were (1) to investigate the effect of the extrusion variables (barrel temperature, feed moisture content, and feed rate) on the physical properties and nutritional components of the extruded products from whole BGW flour; and (2) to optimise the processing conditions for the production of extruded flour products from whole BGW by RSM.

## 2. Materials and Methods

### 2.1. Materials and Sample Preparation

The black-grained wheat (BGW) breeding lines Xinongheidasui and white-grained wheat (WGW) variety Pubing 9946 used in this study were planted during the 2012–2013 crop seasons in the research unit of the North Campus, Northwest A&F University, Yangling, Shaanxi, China (34°20′ N, 108°24′ E, elevation: 526 m a.s.l.). After being harvested, the seeds of two genotypes of wheat were preserved in a small-scale cold storage at 4 °C. The seeds were washed twice using fresh water and dried in a baking oven (FX-11, Saisida, Guangzhou, China) at a low temperature of less than 55 °C. The wheat seed sample was ground into whole-wheat flour with a high-speed multifunctional grinder (JP-1500-8D, Yongkang Jiupin Industry and Trade Co., Ltd., Yongkang, China) to pass through 60-mesh sieves (0.25 mm). The whole-wheat flour samples were stored in sealed plastic bags at 4 °C before the moisture content was set and extrusion was conducted. The whole-grain flours of the WGW were used as the control.

### 2.2. Experimental Design

The research consisted of two parts:

In the first part of the study, three single-factor experiments with three replicates was designed. The effects of the extrusion variables (barrel temperature, feed moisture content, and feed rate) on the physical properties and the nutritional components of the whole BGW flour extrudates were investigated. In the first experiment, the barrel temperature was changed from T1 to T5 (barrel temperature in zones 1–5), while the feed moisture content and the feed rate were fixed at 20.0% and 40 g·min^−1^, respectively (Table 1). In the second experiment, the barrel temperature and the feed rate were fixed at T3 and 40 g·min^−1^, respectively, while the feed moisture content was varied as 15.0%, 17.5%, 20.0%, 22.5%, and 25.0%. In the third experiment, the feed rate was adjusted as follows: 20.0, 30.0, 40.0, 50.0, and 60.0 g·min^−1^, while the barrel temperature and the feed moisture content were fixed at T3 and 20.0%, respectively. The range of independent variables was established on the basis of previous studies [47,48] and preliminary trails. 

In the second part of the study, a three-level, three-variable Box–Behnken design was used in the RSM analysis to optimise the processing conditions for the extrusion cooking of whole BGW flour. The independent variables were barrel temperature (average barrel temperature in zones 1–5) (*X*_1_), feed moisture content (*X*_2_), and feed rate (*X*_3_), which were coded at the levels of −1, 0, and +1. The range of variables was established on the basis of the single-factor experiments. The actual values of the variation levels and the experimental design for this part of the study are shown in Table 2. The dependent variables were specific volume, expansion ratio, hardness, fracturability, WAI, and WSI.

The moisture content of the whole-wheat flour was determined according to Approved Method 44-16 (AACC International 2000). The moisture content was expressed as the wet basis. Whole-wheat flour was mixed with distilled water to reach the designed moisture content. The water addition amount of 100 g of raw material was calculated using the following formula:

Water addition amount (mL·100 g−^1^) = (Designed moisture content − raw material moisture content)/(100 − designed moisture content) × 100. (1)

The moisture content of the whole BGW flour and the WGW flour was 11.7% and 10.8% (w/w), respectively. The conditioned raw samples were stored in sealed plastic bags for 2 h before extrusion.

The feed rate was determined by a change in the screw speed, which was controlled by the computerised control and data acquisition system. Therefore, the feed rate was exchanged from weight per minute (g·min^−1^) to screw speed per minute (r·min^−1^), by using the double helix volume metering feeder, for the veracity and the manoeuvrability of the experiments. The screw speed corresponding to the feed rate is displayed in Table 3.

### 2.3. Extrusion Cooking

Extrusion was carried out using a DSE-25 twin-screw extruder (Brabender, OHG, Duisburg, Germany), equipped with a computer control and data acquisition system. The raw materials were fed into the extruder barrel having a diameter of 25 mm and a length of 600 mm, which consisted of five independent zones. Then, the screw conveyed the material along the barrel. With a further movement of the barrel, the smaller thread-depths hindered the volume and increased the resistance to the movement of the material. The material was subsequently filled in the barrel space between the screw threads and was compressed. As it moved down further along the barrel, the screw kneaded the material into a semi-solid plasticised material. Eventually, with the squeeze, the extrudates were passed through the die at the discharge end of the barrel. The material was collected when the operation condition was steady, as determined by the value of the torque and the system that varied less than 5%. The transition section temperature among the five zones was controlled by electrical heating and compressing water and air cooling. A computerised control and data acquisition system was used to adjust the five set temperatures. The frequency of date acquisition was every 10 s. Each setting was repeated three times. The diameter and the length-to-diameter ratio (*L/D*) of the screw were 25 mm and 20:1, respectively. The screw configuration consisted of conveying element (length with 12.5 mm, helix angle with −27°, screw distance with 29 mm, screw groove width with 13 mm, *D1* with 25 mm, *D2* with 17.5 mm, 2 threads), kneading elements (length with 37.5 mm, oriented at 45° feed forward, kneading pan with 7.5 mm, 5 kneading pans), and gear-type elements (length with 37.5 mm, tooth width with 3.0 mm, gap width with 4.3 mm). Moreover, a constant screw speed of 200 r·min^−1^ was used and a die with a diameter of 6 mm for all the experiments was conducted in this study.

Approximately 300 g of the extrudates was taken and manually cut into 20-cm-long pieces, cooled for 15 min at room temperature, and kept in sealed plastic bags at room temperature. The extrudates were then dried at 103 °C in a baking oven (FX-11, Saisida, Guangzhou, China) to a constant weight. The dried extrudates were ground (particle size < 0.25 mm) and subjected to a functional and nutritional analysis.

### 2.4. Chemical Composition, Physicochemical Properties, and Texture Profile Analysis

#### 2.4.1. Specific Volume

The weight of the extrudate samples was obtained using a DS-671 electronic scale. The extrudate samples’ volume was measured by millet displacement. The specific volume was calculated as a ratio of the volume and the weight of the extrudate samples.

#### 2.4.2. Expansion Ratio

The extrudate samples were dried in a baking oven (FX-11, Saisida, Guangzhou, China) (45 °C) to measure their diameters with mechanical callipers (601-01S, Links HMCT Group, China). The expansion ratio was calculated as the average measurement of 10 extrudate samples’ diameters divided by the die diameter of 6 mm.

#### 2.4.3. Hardness and Fracturability

The hardness and the fracturability of the extruded samples were measured using a TA-XT PLUS texture analyser a 490 N load cell (HDP/3PB, Stable Micro Systems, Godalming, UK). The dried samples with a length of 10 cm were fixed to have a spacing of 3 cm between the horizontal support arms and were pressed down with a knife-edge probe until they broke into half. The testing conditions were as follows: speed of 1.0 mm/s, 1.0 mm/s, and 10.0 mm/s before, during, and after the test, respectively; trigger force of 0.049 N; distance of 5 mm; and the compressed test mode. Hardness was evaluated by calculating the maximum force and evaluated fracturability by measuring the contact distance of the break. The smaller the absolute value of the fracture contact distance was, the greater was the fracturability. Each sample was tested 10 times. The average value was calculated after removing the maximum and the minimum values.

#### 2.4.4. Water Absorption Index (WAI) and Water Solubility Index (WSI)

The WAI and the WSI of the whole-wheat flour samples extruded under different conditions was measured according to the method developed by Balasubramanian et al. [48], with minor modifications. Here, 2.0 g of the ground extrudates was combined with 25 mL of distilled water in a beaker at room temperature while gently stirring for 30 min with a magnetic stirrer (86-1, Sile, Shanghai, China). Then, 32.5 g of the suspensions was transferred to a 50-mL round-bottomed centrifuge tube, which then was centrifuged at 4500× *g* for 15 min (HC-3018, Zhongkezhongjia, Anhui, China). The supernatant was decanted into a dish and evaporated to dryness at 105 °C until a constant weight was reached. Five measurements were performed for each treatment and averaged. The WAI and WSI were calculated using the following formulas:

WAI% = weight of sediment/dry weight of sample × 100.(2)

WSI% = weight of dried supernatant/weight of dry sample × 100.(3)

#### 2.4.5. Protein Content

Nitrogen (N) concentration was measured using the Kjeldahl nitrogen determination method (AACC-approved method 46–13) with an automatic nitrogen determination analyser (Kjeltec 8400, FOSS). The protein content was calculated as follows:

Protein content % (in dry basis) = N × 5.7 × 100.(4)

#### 2.4.6. Ash Content

Ash content was determined by the dry combustion of 3 g of an extrudate sample in a muffler furnace (Jujing, Shanghai, China) at 580 °C for 16 h (AACC approved method 08–01).

#### 2.4.7. Microelements Content

The microelements content—iron (Fe), zinc (Zn), copper (Cu), and manganese (Mn)—of the extrudate samples was determined using flame atomic absorption spectrometry, with a 2000 polarisation Zeeman atomic absorption spectrometer with hollow cathode lamps (Hitachi, Tokyo, Japan), to quantify the aqueous constituents following microwave digestion with an HNO_3_–H_2_O_2_ solution according to the Standard Method GB/T 5009.14-2017 developed by the Ministry of Health of China.

#### 2.4.8. Total Starch Content

Total starch content was evaluated by AACC Approved Method 76-13.

### 2.5. Statistical Analysis

The mean values for the physical properties and the nutritional component parameters were calculated in the whole BGW and WGW flour extrudates. The data were recorded as the means ± standard deviation (SD). Statistical significance was detected by an analysis of variance (ANOVA) using the JMP V12.0 statistical software from SAS (version 9, SAS Institute Inc., Cary, NC, USA). To investigate the effects of the variable levels (barrel temperature, feed moisture content, and feed rate) on the quality of the extrudate made with different coloured wheat flour, significant differences between different variable levels and between the whole BGW and WGW flour extrudates were detected using Fisher’s protected LSD at α = 0.01 and α = 0.05, respectively.

The RSM analysis was completed using Design-Expert version 10.0.4 (Statease Inc., Minneapolis, MN, USA). A multiple linear regression method of the second-order polynomial model was performed using the experimental data to fit to the selected models and regression coefficients, obtained from the same software. The generating regression equation can be expressed as follows:

Y = α_0_ + α_1 *×* 1_ + α_2_*X*_2_ + α_3_*X*_3_ + α_4_*X*_1_*X*_2_ + α_5_*X*_1_*X*_3_ + α_6_*X*_2_*X*_3_ + α_11_*X*_1_^2^ + α_22_*X*_2_^2^ + α_33_*X*_3_^2^. (5)

where Y is the experimental response; α_0_ is the constant; α_1_, α_2_, and α_3_ are the linear coefficients; α_4_, α_5_, and α_6_ are the interaction coefficients; α_11_, α_22_ and α_33_ are the squared coefficients; and *X*_1_*, X*_2_ and *X*_3_ are the barrel temperature, feed moisture content and feed rate, respectively. The statistical significance of the terms was examined in the regression equation by using an ANOVA for each response. The adequacy of the models was determined by *R*^2^ and lack-of-fit tests (*p* < 0.05). The desirability function of the RSM was executed to optimise the independent variables of the extrusion processing of whole BGW flour. Furthermore, three-dimensional (3D) surface plots were generated as a function of two independent variables when the third independent variable was kept constant from the models in the same software.

## 3. Results and Discussion

### 3.1. Effect of Barrel Temperature on Extrudate Quality

The extrusion temperature affected the expansion volume of the extrudates [50]. The effects of the barrel temperature on the physical properties and the nutritional components of the wheat extrudates are shown in Table 4. The specific volume and the expansion ratio of the extrudates were significantly decreased with an increase in the barrel temperature (α = 0.01), indicating that the increasing barrel temperature had an adverse effect on the specific volume and the expansion ratio of the whole-wheat flour extrudates. A similar observation was reported for barley flour and corn starch [33,38]. The expansion ratio decreased because of the decrease in the viscosity of the starches brought about by the increasing barrel temperature [38]. The WAI of the extrudates was used to measure the amount of water held by the starch after its dispersion in excess water, which might have been associated with the degree of starch damage because of its gelatinisation and fragmentation during extrusion under high-temperature conditions [51]. The WSI of the extrudates was used to determine the amount of the soluble components released from the starch during extrusion, which was an indicator of the degradation of the molecular components [52]. The WAI and the fracturability of the extrudates were increased by increasing the barrel temperature (T1–T3) and decreased with further increases in the barrel temperature (T3–T5), whereas the hardness showed the opposite trend (Table 4). The increase in the WSI of the extrudates with increasing barrel temperature was attributed to the increase in soluble components with starch melting by extrusion [53,54]. The results were in agreement with those reported by Ding et al. [47], who stated that a higher zone-5 barrel temperature may increase the degree of wheat starch gelatinisation, thereby lowering the WAI and increasing the WSI of the extrudates. A reduction in WAI and an increase in WSI with an increase in the temperature (125–135 °C) was reported [55]. The fracturability of the extrudates was the highest under the T3 conditions. No significant differences in hardness were found between the T2 and the T3 conditions, and no such differences in WAI and WSI were found among the T2, T3, and T4 conditions. The increasing barrel temperature did not affect significantly the protein, ash, Fe, Zn, Cu, and Mn content of the extrudates (Table 4), whereas it reduced significantly the total starch content of the extrudates (α = 0.01) (Table 5). Extrusion modifies the crude protein distribution but does not alter its content [56]. The non-significant variation in the protein content is in accordance with previous studies [57,58]. The increasing temperature promotes protein unfolding, leading to protein re-association [59]. The possible reason for no significant changes in the protein content could be attributed to the high temperature promoting the formation of isopeptides [60]. A similar finding has been reported, wherein the temperature (140, 160, and 180 °C) did not considerably affect the Fe and Zn availability of the extruded bean flour [36]. However, there was a significantly higher content of Fe and Cu in the extrudates (α = 0.01) than in the unextruded flour. Extrusion can cause the destruction of anti-nutritional components [44]. During extrusion, the heat-labile compounds, such as the phytic acid, trypsin inhibitor, and tannins, were significantly reduced by the combination of the thermal treatment and the mechanical shear of the screw speed, enhancing the nutritional value [61]. Chen et al. [18] reported phenolic acids (except for gallic acid) and flavonoid of Jizi439 black wheat bran were significantly increased by optimizing the extrusion and extraction condition. This result may be attributed to the destruction of anti-Fe and Cu nutritional factors of wheat by extrusion [54]. Native starch content is altered due to the applied temperature and presence of water content during extrusion. Two basic processes can occur in these circumstances: starch degradation and starch polymerization [62]. This could be the reason for the decreasing trend in the total starch in this study.

The effects of the barrel temperature on the extrudate quality were further compared between two wheat genotypes (Table 4 and Table 5). There were no significant differences in the specific volume, expansion ratio, hardness, fracturability, and WSI between the extrudates of the two wheat genotypes under the T2, T3, T4, and T5 conditions. The WAI of the BGW extrudates was significantly lower than the WGW extrudates under the five temperature levels (α = 0.05), which could be attributed to the higher protein content of flour for BGW (16.53% versus 14.24%) (Table 4). The result of the WAI is confirmed by a previous study [63], which reported a significant negative relationship between the WAI and kernel protein content. The value of WAI highly depends on the nature of the proteins. The WAI value of hemp protein concentrate was lower than that of soy protein isolates [64].

Microelements are a crucial nutrient required for many physiological functions in humans. The Zn and Fe content in wheat grains in the major production areas in China were in the range of 30.3–30.4 mg⋅kg^−1^ and 45.2–48.2 mg⋅kg^−1^, respectively [3]. The Zn and Fe content in the wheat grains grown in Southern Brazil were insufficient to meet the human daily demand, whereas the Cu and Mn content met the daily requirements [5]. More than half of the worldwide population suffers from micronutrient deficiencies, particularly Zn and Fe deficiencies, because their daily diet depends on cereal crops [65]. The Fe content of refined flour, whole-wheat flour, and wheat bran is 1.17 mg⋅100 g^−1^, 3.86 mg⋅100 g^−1^, and 10.75 mg⋅100 g^−1^, respectively [30]. Whole-wheat flour has been reported to be related to a reduced risk of disease and incidence of multiple noncommunicable diseases [30]. The BGW extrudates had a significantly higher content of protein, ash, Zn (37.06–38.73 mg⋅kg^−1^), Cu, and Mn than the WGW extrudates did under the T1, T2, and T3 conditions, whereas a significantly lower content of Fe (43.54–47.32 mg⋅kg^−1^) and total starch was found in the BGW extrudates (α = 0.05).

These results showed that the barrel temperature for the optimisation of the whole BGW extrudates was considered in the range of T2 to T4.

### 3.2. Effect of Feed Moisture Content on Extrudate Quality

The moisture content in the raw materials leads to gelatinisation reactions, which in turn affect the physical properties and the nutritional components of the extrudates. The degree of starch conversion was reduced by the increase in the feed moisture content [35]. The low feed moisture content resulted in the dextrinisation increase during extrusion because of the relatively high viscosity and shear stress [66]. As indicated in Table 6, the specific volume, expansion ratio, and fracturability of the extrudates were increased with an increase in the feed moisture content from 15.0% to 20.0% and declined with a further increase in the feed moisture content, but the hardness had the opposite tendency. The increase in the specific volume and the expansion rate of starch with an increase in the feed moisture could be attributed to its higher degree of gelatinisation at this feed moisture content (15.0–20.0%) [66], and the reduction in expansion could be attributed to the molecular degradation according to the reports in [35,47]. A WAI increase and WSI decrease were found in the extrudates when the feed moisture content increased (Table 6). This was not consistent with the findings of Ding et al. [47], with a lower WAI and higher WSI upon an increase in the feed moisture content (14–22%), and was slightly different from that reported by Gandhi et al. [38], wherein both WAI and WSI increased with an increase in the feed moisture content in the range of 17–18%. The results in this study could be attributed to the fact that the high feed moisture content (20.0–25.0%) reduced the shear forces, thereby promoting starch dextrinization [34]. The obtained different results may be due to the different materials. It was uncertain which type of wheat was used in the study of Ding et al. [47], who only mentioned wheat flour, while Gandhi et al. [38] used native corn and potato starch. A significantly high specific volume, expansion ratio, and fracturability, and the lowest hardness, were found in the extrudates under the 20.0% conditions (α = 0.01) (Table 6). No significant differences in the WAI and WSI were found for the extrudates between 20.0% and 22.5% and 17.5% and 20.0%, respectively. The protein, ash, Zn, Fe, Cu, and Mn content of the extrudates was not significantly affected by increasing the feed moisture content (Table 6), while the total starch content of the extrudates was significantly reduced (α = 0.01) (Table 5). A similar observation was reported, wherein the Fe and Zn availability of the extruded bean flour did not change considerably with an increase in the moisture content (17–23%) [36]. Similar to the effect of the barrel temperature on the Fe and Cu content, a significantly higher content of Fe and Cu was found in the extrudates under different levels of feed moisture content than in the unextruded flours (α = 0.01).

The effect of the feed moisture content on the extrudate quality was evaluated between the two wheat genotypes (Table 5 and Table 6). A significantly lower specific volume and WAI were found in the BGW extrudates under 20.0%, 22.5%, and 25.0% moisture content conditions than in the WGW extrudates (α = 0.05). The BGW extrudates had a significantly higher expansion ratio than the WGW extrudates under the feed moisture content of 15.0%, 17.5%, and 20.0% (α = 0.05). Under the 20.0% condition, the BGW extrudates had a significantly higher WSI than the WGW extrudates (α = 0.05). No significant differences in the hardness and the fracturability were observed between the extrudates of the two wheat genotypes under the conditions of 15.0%, 17.5%, and 20.0%, and five levels of feed moisture content, respectively. The protein, ash, Zn, Cu, and Mn content of the BGW extrudates was significantly higher than those of the WGW extrudates under the feed moisture content of 20.0%, 22.5%, and 25.0%, but a significantly lower content of Fe (42.76–43.97 mg⋅kg^−1^) and total starch was found in the BGW extrudates (α = 0.05).

These results indicated that the range of the feed moisture content was from 17.5% to 22.5% for the optimisation of the whole BGW extrudates.

### 3.3. Effect of the Feed Rate on Extrudate Quality

As shown in Table 7, the specific volume, expansion ratio, and fracturability of the wheat extrudates increased as the feed rate increased from 20.0 to 40.0 g·min^−1^ and decreased with further increases in the feed rate, whereas the hardness showed an opposite trend. Increasing the feed rate (20–32 kg⋅h^−1^) led to a higher hardness of the extrudates [47]. The screw speed of the extrusion could effectively release cellulosic microcrystals in the cell wall structure that caused the wall to break [45]. The decrease in the specific volume and the expansion ratio with a low feed rate (above 40.0 g·min^−1^) could be attributed to the high flow rate, decreased residence time, and the viscosity of the molten wheat, thereby resulting in not sufficiently broken or degraded macromolecular substances. Changes in the WAI and WSI in wheat extrudates were observed when the feed rate increased from 20.0 to 50.0 g·min^−1^, but no significant difference was found at the feed rates of 20.0, 30.0, 40.0, and 50.0 g·min^−1^. Significantly high specific volume, expansion ratio, and fracturability of the BGW extrudates were observed at the feed rate of 40.0 g·min^−1^ (α = 0.01). There were no significant differences in the hardness, WAI, and WSI of the extrudates at the feed rates of 30.0, 40.0, and 50.0 g·min^−1^. The increasing feed rate had no significant effect on the WSI and the protein, Fe, Zn, Cu, and Mn content of the extrudates (Table 7), but it decreased significantly the total starch content of the extrudates (α = 0.01) (Table 5). Similar to the effect of the barrel temperature and the feed moisture content on the Fe and Cu content, the extrudates obtained under different feed rates had a significantly higher content of Fe and Cu than the unextruded flour (α = 0.01), suggesting that extrusion cooking could improve the nutritional components of a product of whole-wheat flour.

The effect of the feed rate on the extrudate quality was further investigated between the BGW and the WGW extrudates (Table 5 and Table 7). The BGW extrudates had a significantly lower specific volume and WAI than the WGW extrudates at the feed rate of 40.0 g·min^−1^, whereas significantly higher expansion ratio and WSI were found in the BGW extrudates (α = 0.05). Similarly, under three levels of barrel temperature and food moisture content, the BGW extrudates had a significantly higher content of protein, ash, Zn, Cu, and Mn, and significantly lower total starch content than the WGW extrudates at the feed rate with three levels (α = 0.05). Moreover, a higher content of protein, ash, Zn (37.54 mg⋅kg^−1^), Cu (6.02 mg⋅kg^−1^), and Mn (48.67 mg⋅kg^−1^), and lower total starch content (57.50%), were observed in the BGW unextruded flour compared to the WGW unextruded flour. These results showed that the extruded and unextruded flours made of BGW would be available to the consumers as nutritious and functional foods.

These results showed the feed rate for the optimisation of whole BGW extrudates varied between 30.0 and 50.0 g·min^−1^.

### 3.4. Diagnostic Checking of the Fitted Model and Surface Plots for Various Responses

As showed in Table 4, the specific volume and expansion ratio of the extrudates were high under the T1 and T2 treatments, while the lowest hardness and the highest fracturability and WAI were observed for the extrudates under the T3 treatments. The extrudates had the highest specific volume, expansion ratio, and fracturability, and the lowest hardness at a 20.0% feed moisture content (Table 6) and a feed rate of 40.0 g·min^−1^ (Table 7), respectively. The increasing barrel temperature, feed moisture content, and feed rate had no significant effect on the content of protein, ash, Fe, Zn, Cu, and Mn of the extrudates (Table 4, Table 6 and Table 7). Therefore, the Box–Behnken design experiment was conducted with the barrel temperature (T3, 148 °C, average barrel temperature in zones 1–5), feed moisture content (20.0%), and feed rate (40.0 g·min^−1^) as the experimental centre points, and the specific volume, expansion ratio, hardness, fracturability, WAI, and WSI as the response variables (Table 2).

An appropriate response surface model was identified and fitted for the optimal processing conditions of the whole BGW flour products by using RSM. Multiple linear regression equations of the second-order polynomial model were generated with coded variables. An ANOVA was carried out to evaluate the significant effects of the independent variables on various responses and to determine which of the responses were significantly affected by the changing processing conditions.

#### 3.4.1. Specific Volume

The regression equation relating the response function specific volume was represented in terms of the coded variables:

Specific volume = 3.19 − 0.76*X*_1_ − 0.21*X*_2_ + 0.06*X*_3_ − 0.01*X*_1_*X*_2_ − 0.03*X*_1_*X*_3_ + 0.01*X*_2_*X*_3_ + 0.43*X*_1_^2^ − 0.36*X*_2_^2^ − 0.42*X*_3_^2^. (6)

**Figure 1:**
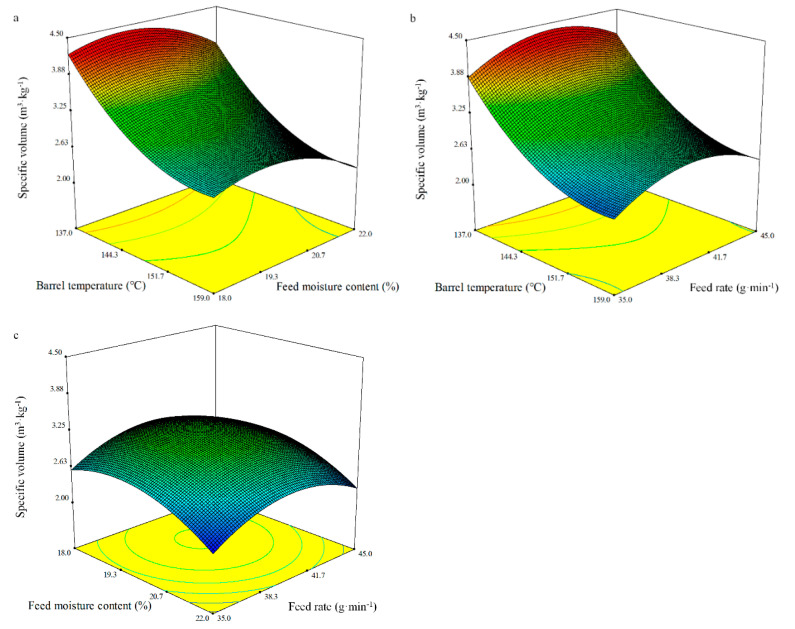
Response surface plot of a specific volume of the whole BGW extrudates.

The surface response was assessed by ANOVA, and the data are presented in Table 8. The quadratic model on specific volume had an excellent fit with a coefficient of determination (*R*^2^) of 0.98 for the whole BGW extrudates. The model as fitted was significant (*p* < 0.0001), whereas the lack of fit was not significant. The regression analyses showed that the specific volume was significantly dependent on the linear (*p* < 0.0001, *p* < 0.0001, and *p* < 0.01, respectively) and quadratic (*p* < 0.0001) terms of the barrel temperature (*X*_1_), feed moisture content (*X*_2_), and feed rate (*X*_3_) (Table 8). The values for the specific volume of the whole BGW extrudates were in the range of 2.14 to 4.25 m^3^·kg^−1^ (Figure 1). The 3D surface plot shows that the specific volume increased with an increase in the feed moisture content and the feed rate before it reached a critical feed moisture content and feed rate after which it decreased, which may be due to starch dextrinization and a weakened structure [44]. The materials is unable to melt and gelatinize sufficiently when the moisture content is too low, while the shear and friction on the materials are significantly reduced by the water’s lubrication function when the moisture content is too high, thus leading to a decrease in the specific volume [50]. When the feed rate is small, the shear and friction on the material is not enough, which leads to shrivelled extrudates. When the feed rate increases, the residence time of the materials decreases in the barrel, which results in inadequate time to absorb enough heat, and thus extruding a bad quality extrudate. With the increase in the barrel temperature, the specific volume dropped sharply in this study, which was not in accordance with the finding of Wu [67] with the same effect of the feed moisture content and feed rate on the specific volume of the whole BGW extrudates. It may be due to changes in the barrel temperature in zones 2 to 5 (Table 1).

#### 3.4.2. Expansion Ratio

The expansion ration of the extruded materials is an important parameter linked to the interaction between starch and protein [56]. The expansion ratio of flour products depends on the atmospheric pressure, water vapour pressure, and the capacity of the product to sustain expansion. The effect of the barrel temperature (*X_1_*), feed moisture content (*X_2_*), and feed rate (*X_3_*) on the expansion ratio can be described using Equation (7) in terms of the coded values:

Expansion ratio = 0.93 − 0.17*X*_1_ − 0.08*X*_2_ + 0.03*X*_3_ − 0.02*X*_1_*X*_2_ − 0.01*X*_1_*X*_3_ + 0.01*X*_2_*X*_3_ + 0.09*X*_1_^2^ − 0.09*X*_2_^2^ − 0.10*X*_3_^2^. (7)

**Figure 2:**
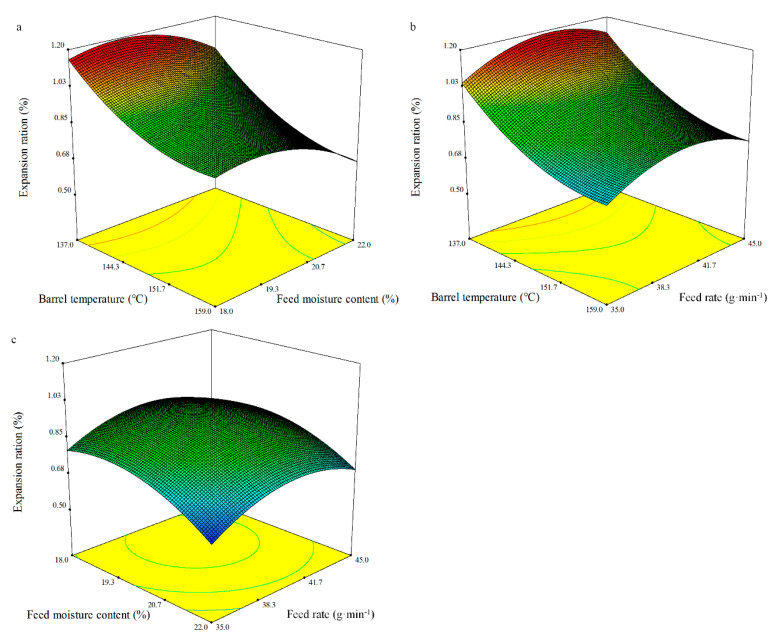
Response surface plot of the expansion ratio of the whole BGW extrudates.

The ANOVA results for the regression models of the expansion ratio are shown in Table 8. The fitted model had a good coefficient of determination value (*R*^2^ = 0.96) for the expansion ratio of the extrusion of whole BGW flour. The model of the expansion ratio as fitted was significant (*p* < 0.0001), and the lack of fit was not significant. The regression analyses indicated that the expansion ratio was significantly affected by the linear terms of the barrel temperature (*X_1_*) (*p* < 0.0001), feed moisture content (*X_2_*) (*p* < 0.01), and the feed rate (*X_3_*) (*p* < 0.05), and the quadratic terms of the barrel temperature (*X_1_*), feed moisture content (*X_2_*), and feed rate (*X_3_*) (*p* < 0.001). This result is somewhat consistent with the findings of Nyombaire et al. [68], in which only feed rate had a significant effect on the expansion ratio of kidney beans among all the extrusion variables. This lack of effect may be caused by the high protein content of red kidney beans compared to cereal grains. However, the feed rate (20–32 kg·h^−1^) did not significantly affect the expansion ratio as reported by Ding et al. [47]. The different result may be due to the extrusion conditions and the material used. With increasing barrel temperature, the expansion ratio declined sharply (Figure 2), which is in agreement with a negative relationship between higher temperatures and the expansion ratio [58]. The expansion ratio of the whole BGW extrudates was between 0.60 and 1.16% (Figure 2).

#### 3.4.3. Hardness

Multiple regression equations for hardness as a function of the barrel temperature (*X_1_*), feed moisture content (*X_2_*), and feed rate (*X_3_*) are described in terms of the coded variables as follows:

Hardness = 3.84 − 1.86*X*_1_ + 0.20*X*_2_ − 0.45*X*_3_ − 0.32*X*_1_*X*_2_ + 0.13*X*_1_*X*_3_ − 0.095*X*_2_*X*_3_ + 0.82*X*_1_^2^ + 0.38*X*_2_^2^ + 0.75*X*_3_^2^. (8)

**Figure 3:**
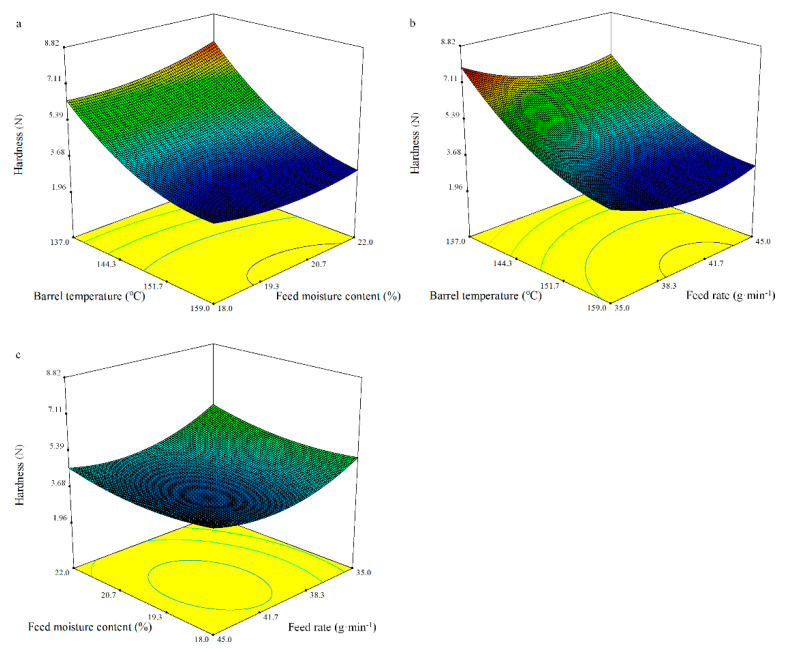
Response surface plot of the hardness of the whole BGW extrudates.

The model as fitted indicated significance (*p* < 0.0001) with no significant lack of fit (Table 8). A high coefficient of determination (*R*^2^) of 0.97 was observed for the model of hardness as fitted. This showed that the barrel temperature (*X_1_*) and the feed rate (*X_3_*) had a significant negative linear effect (*p* < 0.0001 and *p* < 0.01, respectively), whereas its significant quadratic effect was positive (*p* < 0.001). Those effects have been reported previously [47]. The values for hardness in the extrusion cooking of BGW flour widely varied from 3.18 to 7.72 N (Figure 3). 

#### 3.4.4. Fracturability

The predicted model for fracturability can be represented in terms of the coded values by the following equation:

Fracturability = −0.98 + 0.12*X*_1_ − 0.21*X*_2_ + 0.14*X*_3_ + 0.01*X*_1_*X*_2_ − 0.02*X*_1_*X*_3_ + 0.01*X*_2_*X*_3_ − 0.24*X*_1_^2^ − 0.17*X*_2_^2^ − 0.44*X*_3_^2^. (9)

**Figure 4:**
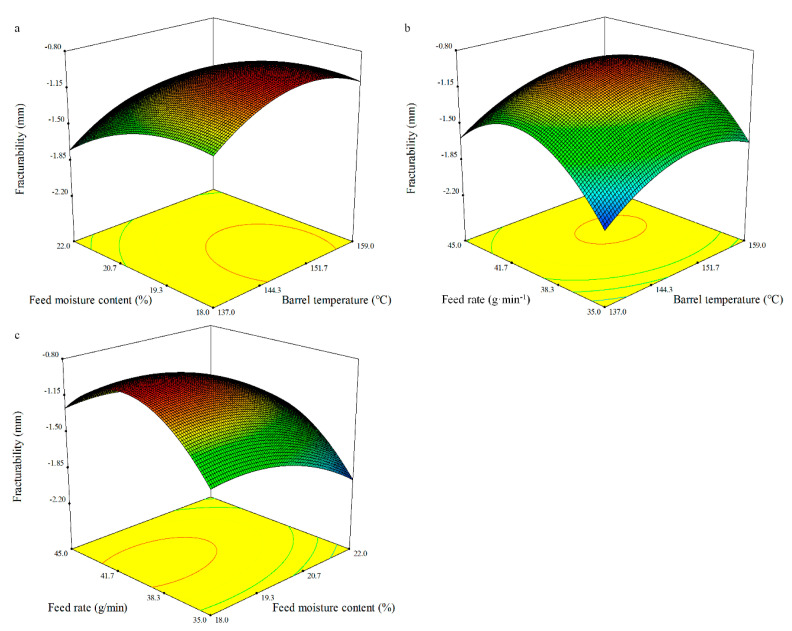
Response surface plot of the fracturability of the whole BGW extrudates.

The ANOVA showed the quadratic model of fracturability was significant (*p* < 0.0001) and the lack of fit was not significant (Table 8). The regression model for the effect of the independent variables on the fracturability of the whole BGW flour extrudates had a high coefficient of determination (*R*^2^) of 0.97. The fracturability of the extrudates was significantly dependent on the linear terms of the barrel temperature (*X_1_*), feed moisture content (*X_2_*), and feed rate (*X_3_*) (*p* < 0.001, *p* < 0.0001, and *p* < 0.001, respectively), and the quadratic terms of the barrel temperature (*X_1_*), feed moisture content (*X_2_*), and feed rate (*X_3_*) (*p* < 0.0001, *p* < 0.001, and *p* < 0.0001, respectively), which are in agreement with the finds of Badrie et al. [69]. The value of fracturability was between −0.92 and −2.02 mm (Figure 4).

#### 3.4.5. WAI

The regression equation related to the response function WAI can be expressed in terms of the coded variables as follows:

WAI = 6388.68 + 212.54*X*_1_ + 168.17*X*_2_ − 28.73*X*_3_ − 103.51*X*_1_*X*_2_ + 18.97*X*_1_*X*_3_ + 8.45*X*_2_*X*_3_ − 409.17*X*_1_^2^ − 166.44*X*_2_^2^ − 62.13*X*_3_^2^. (10)

**Figure 5:**
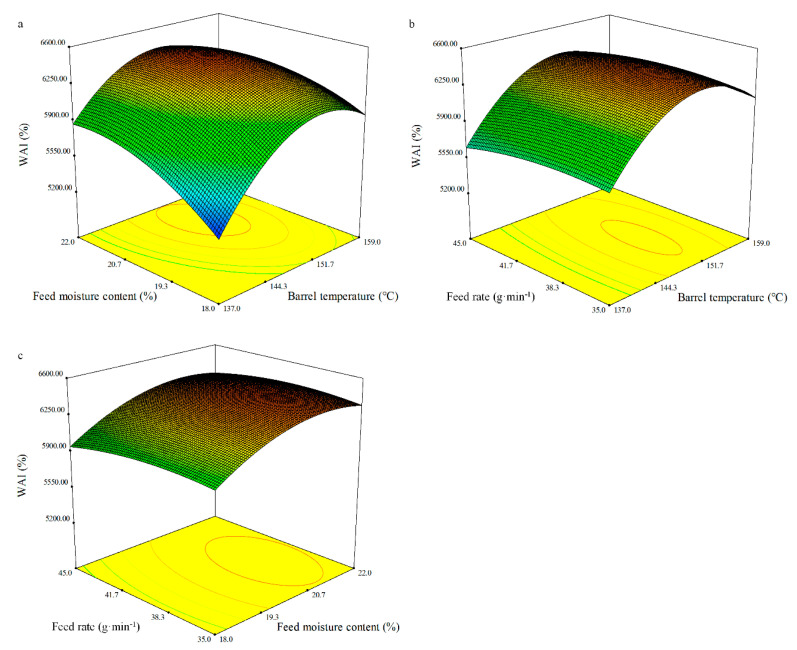
Response surface plot of the WAI of the whole BGW extrudates.

The ANOVA showed that the quadratic model of WAI as fitted was significant (*p* < 0.01), and the lack of fit was not significant (Table 8). An acceptable coefficient of determination (*R*^2^) of 0.83 was obtained for the response surface regression models on WAI. WAI was highly significant for the linear terms of the barrel temperature (*X_1_*) and the feed moisture content (*X_2_*) (*p* < 0.01), and the quadratic terms of the barrel temperature (*X_1_*) (*p* < 0.001) and the feed moisture content (*X_2_*) (*p* < 0.05). The feed rate did not significantly affect the WAI. The measured WAI of the whole BGW flour extrudates varied between 5261.32 and 6543.47% on the basis of the level of the extrusion variables (Figure 5). WAI describes the amount of water held by the extrudates and is mainly triggered by the gelatinization and melting of molecules [56]. The response surface showed that the barrel temperature and the feed moisture content had a dominant effect on the WAI, whereas the feed rate seemed to have a minor effect. Although the feed rate was not a significant independent variable, a slight increase in the WAI with an increase in the feed rate was found (Figure 5). The battle temperature is the most important processing variables as it highly influences protein conformational changes [56], while protein denaturation occurring under high feed moisture conditions is the main phenomena effecting the hydration properties of the extrudates [70]. This could be the reason for the results in the presented study.

#### 3.4.6. WSI

WSI and WAI are the most relevant hydration properties, which explain their behaviour in the presence of water. Extrusion has been proven to contribute to improve the hydration properties of different extrudes. The regression equation for WAI was determined in terms of the coded variables as follows:

WSI = 224.39 − 8.98*X*_1_ − 17.54*X*_2_ + 3.56*X*_3_ − 1.22*X*_1_*X*_2_ − 5.78*X*_1_*X*_3_ + 0.16*X*_2_*X*_3_ − 34.7*X*_1_^2^ − 40.79*X*_2_^2^ − 28.19*X*_3_^2^. (11)

**Figure 6:**
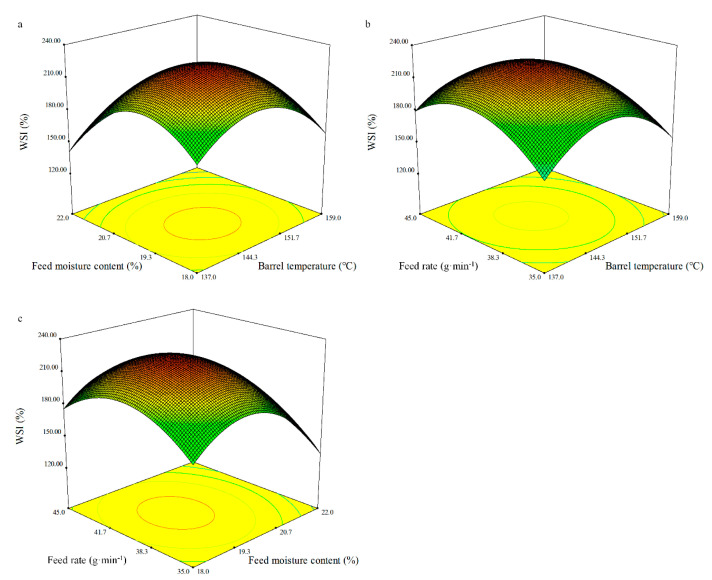
Response surface plot of the WSI of the whole BGW extrudates.

The model of WAI as fitted was observed to be significant (*p* < 0.001), whereas the lack of fit was not significant (Table 8). The coefficients of determination (*R*^2^) for WAI were 0.93. The regression analysis showed that the WSI was significantly affected by the linear terms of the barrel temperature (*X_1_*) and the feed moisture content (*X_2_*) (*p* < 0.05 and *p* < 0.01, respectively), and the quadratic terms of the barrel temperature (*X_1_*), feed moisture content (*X_2_*), and feed rate (*X_3_*) (*p* < 0.001, *p* < 0.0001, and *p* < 0.001, respectively). The WSI of the BGW flour extrudates varied from 123.85 to 231.46% (Figure 6).

### 3.5. Optimisation

**Figure 7:**
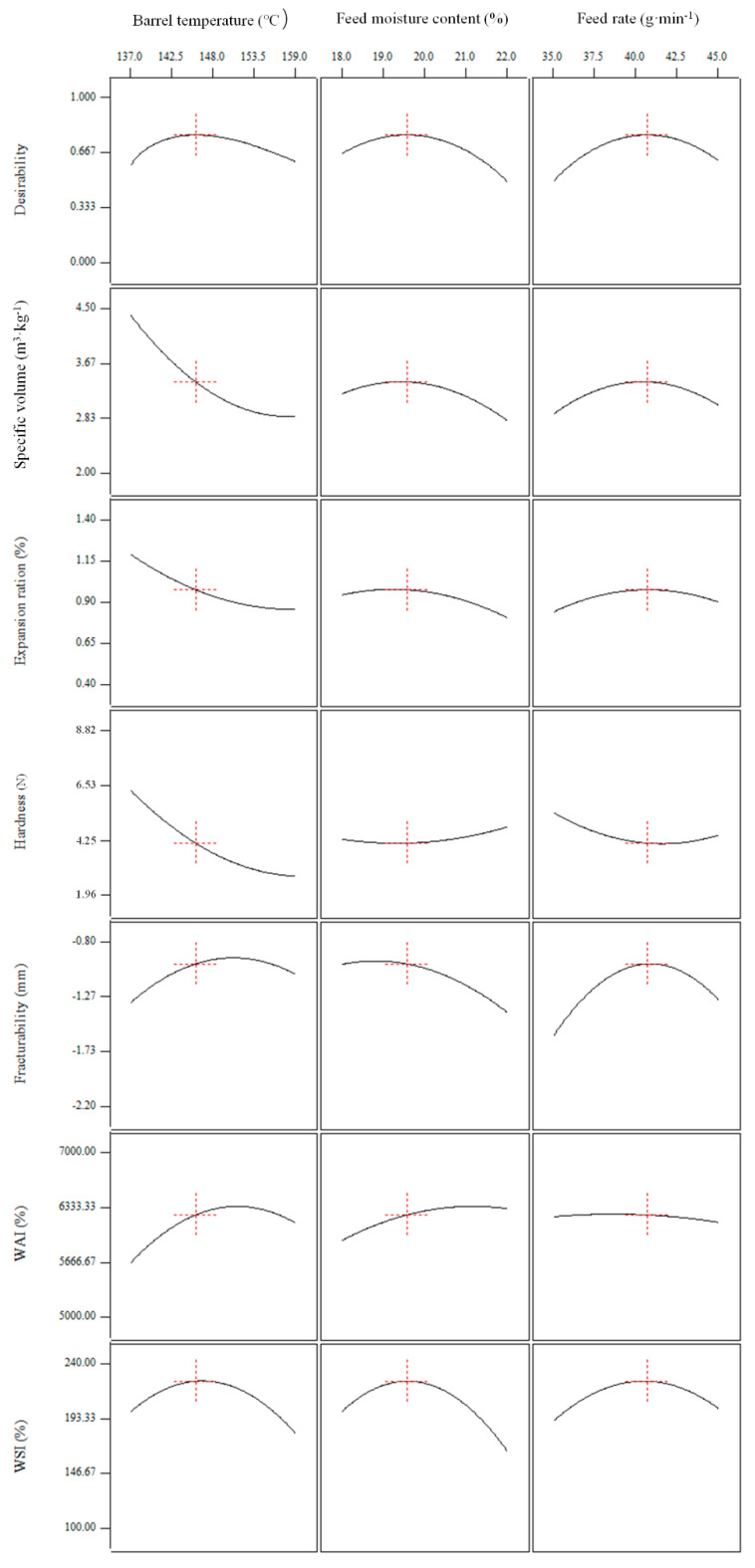
Desirability function response surface of the whole BGW extrudates.

Obtaining the optimum values of a function of certain independent variables subject to certain constraints is called optimisation. The maximum value of a desired dependent variable and the minimum value of an undesired one are given in the optimisation process. The optimum processing conditions were the values of the independent variables, which could generate the desired optimum value [71]. Product responses, including the specific volume, expansion ratio, hardness, fracturability, WAI, and WSI, were important major parameters determining the quality of the extrudates. Therefore, optimum conditions for extrusion of the whole BGW flour were determined to obtain the maximum specific volume, expansion ratio, fracturability, WAI and WSI, and minimum hardness values. To obtain the optimum conditions in the extrusion cooking of the whole BGW flour, a numerical optimisation was performed using the desirability function of the RSM. The desirability function first obtained the maximum and minimum values of each response on the basis of the statistical analysis. Then, the general function optimisation method was applied to determine the optimal setting of the independent variables (within the specified range) for the overall response desirability (Figure 7). After optimisation with the desirability function, the desirability value of 0.781 was obtained. One solution was acquired for the optimum processing conditions to produce the whole BGW extrudates. The optimum barrel temperature, feed moisture content, and feed rate estimated were 145.63 °C, 19.56%, and 40.64 g·min^−1^, respectively. Under the optimum conditions, the desirable whole BGW extrudates had expected values with a specific volume of 3.40 (m^3^·kg^−^^1^), expansion ratio of 0.98 (%), hardness of 4.19 (N), fracturability of −0.97 (mm), WAI of 6268.34 (%), and WSI of 226.70 (%). By applying these optimal conditions, an edible whole BGW extrudate with a specific volume equal to 3.18 (m^3^·kg^−^^1^), expansion ratio of 0.93 (%), hardness of 3.99 (N), fracturability of −1.08 (mm), WAI of 6346.31 (%), and WSI of 217.32 (%) could be produced.

## 4. Conclusions

The properties of the wheat-derived extrudates produced on a twin-screw extruder depended on several process variables. To improve our understanding of how the barrel temperature, feed moisture content, and feed rate affect the physical properties and nutritional components of the extrudates from the BGW flour, and to obtain its optimum extrusion parameters, a BGW breeding line (Xinongheidasui) was used as the sample material and a WGW cultivar (Pubing 9946) was used as the control.

Increasing the barrel temperature, feed moisture content, and feed rate had some significant effects on the physical properties and the nutritional components of the products extruded from whole BGW flour. Extrusion cooking could improve the nutritional components of the product of the whole BGW flour. Extruded and unextruded flours made of whole BGW had better nutritional components than the WGW flours. The extrudates of whole BGW flour could be made available for consumption by health-conscious consumers, or whole BGW flour could be used directly by food-processing companies as a novel food material. The optimal processing conditions included a barrel temperature of 145.63 °C, feed moisture content of 19.56%, and a feed rate of 40.64 g·min^−1^. These conditions led to the best results of the whole BGW extrudates in terms of the specific volume, expansion ratio, fracturability, WAI, WSI, and hardness.

## Figures and Tables

**Table 1:** Temperature profile in the extruder zones.

Treatments	Zones/°C					Average
	1	2	3	4	5	
T1	60	120	140	150	160	126
T2	60	130	155	165	175	137
T3	60	140	170	180	190	148
T4	60	150	185	195	205	159
T5	60	160	200	210	220	170

**Table 2:** Box–Behnken experimental design for extrusion of whole BGW flour.

Run	Coded Variables			Actual Variables		
	*X*_1_	*X*_2_	*X*_3_	Barrel Temperature (°C) ^a^	Feed Moisture Content (%)	Feed Rate (g·min^−1^)
1	−1	0	1	137	20	45
2	−1	−1	0	137	18	40
3	−1	1	0	137	22	40
4	−1	0	−1	137	20	35
5	0	−1	1	148	18	45
6	0	0	0	148	20	40
7	0	0	0	148	20	40
8	0	0	0	148	20	40
9	0	1	1	148	22	45
10	0	−1	−1	148	18	35
11	0	1	−1	148	22	35
12	1	0	1	159	20	45
13	1	−1	0	159	18	40
14	1	1	0	159	22	40
15	1	0	−1	159	20	35

^a^ Extruder barrel temperature in zones 1–5. BGW, black-grained wheat.

**Table 3:** Screw speed corresponding to feed moisture content and feeding rate.

Feed Moisture Content (%)	Feed Rate (g·min^−1^)	Screw Speed (r·min^−1^)	
		BGW	WGW
20.0%	20.0	16.7	12.4
	30.0	20.0	16.7
	40.0	26.0	21.6
	50.0	34.7	27.1
	60.0	46.1	33.2
22.5%	20.0	16.7	12.5
	30.0	23.2	18.6
	40.0	30.7	22.1
	50.0	39.2	28.5
	60.0	51.7	34.6
25.0%	20.0	16.7	12.5
	30.0	22.3	18.3
	40.0	30.7	24.2
	50.0	45.5	30.5
	60.0	63.2	37.3

BGW, black-grained wheat; WGW, white-grained wheat.

**Table 4:** Effect of barrel temperature on the physical properties and nutritional components of the wheat extrudates.

Barrel Temperature (°C) ^a^	Specific Volume (m^3^·kg^−1^)		Expansion Ratio (%)		Hardness (N)	
	BGW	WGW	BGW	WGW	BGW	WGW
T1	5.07 ± 0.21 ^Aa^	4.85 ± 0.06 ^Aa^	1.58 ± 0.06 ^Aa^	1.49 ± 0.03 ^Aa^	7.88 ± 0.36 ^Ab^	8.95 ± 0.49 ^Aa^
T2	4.69 ± 0.17 ^Aa^	4.53 ± 0.09 ^Aa^	1.23 ± 0.04 ^Ba^	1.15 ± 0.04 ^Ba^	4.38 ± 0.43 ^Ca^	4.74 ± 0.51 ^Ca^
T3	3.32 ± 0.06 ^Ba^	3.68 ± 0.03 ^Ba^	1.00 ± 0.08 ^Ba^	0.82 ± 0.07 ^Ca^	3.80 ± 0.55 ^Ca^	4.09 ± 0.32 ^Ca^
T4	2.36 ± 0.06 ^Ca^	2.69 ± 0.04 ^Ca^	0.72 ± 0.04 ^Ca^	0.68 ± 0.08 ^Ca^	5.50 ± 0.45 ^Ba^	6.50 ± 0.51 ^Ba^
T5	1.58 ± 0.04 ^Da^	1.71 ± 0.03 ^Da^	0.56 ± 0.07 ^Ca^	0.42 ± 0.07 ^Da^	7.43 ± 0.59 ^Aa^	8.36 ± 0.63 ^Aa^
	Fracturability (mm)		WAI (%)		WSI (%)	
T1	−1.68 ± 0.08 ^Ba^	−1.89 ± 0.07 ^Cb^	5434.00 ± 70.71 ^Bb^	5647.00 ± 72.33 ^Ba^	195.32 ± 16.21 ^Ba^	134.56 ± 12.93 ^Ca^
T2	−1.31 ± 0.11 ^Aa^	−1.53 ± 0.04 ^Ba^	6168.00 ± 59.40 ^Ab^	6368.00 ± 82.63 ^Aa^	212.82 ± 20.27 ^Ba^	162.34 ± 24.61 ^BCa^
T3	−0.99 ± 0.18 ^Aa^	−0.84 ± 0.03 ^Aa^	6486.00 ± 73.54 ^Ab^	6754.00 ± 84.85 ^Aa^	234.00 ± 28.40 ^Ba^	188.80 ± 18.95 ^Ba^
T4	−1.26 ± 0.09 ^Aa^	−1.43 ± 0.08 ^Ba^	6294.00 ± 68.43 ^Ab^	6523.00 ± 60.32 ^Aa^	238.34 ± 20.21 ^Ba^	200.54 ± 20.12 ^Ba^
T5	−1.76 ± 0.10 ^Ba^	−1.86 ± 0.12 ^Ca^	6063.00 ± 50.42 ^Ab^	6332.00 ± 77.86 ^Aa^	265.25 ± 17.14 ^Aa^	245.32 ± 17.24 ^Aa^
	Protein content (%)		Ash content (%)		Fe content (mg⋅kg^−1^)	
Unextruded	16.53 ± 0.01 ^Aa^	14.24 ± 0.09 ^Ab^	1.71 ± 0.01 ^Aa^	1.53 ± 0.06 ^Ab^	33.56 ± 1.32 ^Bb^	43.76 ± 0.34 ^Ba^
T1	16.45 ± 0.12 ^Aa^	14.34 ± 0.11 ^Ab^	1.73 ± 0.04 ^Aa^	1.55 ± 0.04 ^Ab^	45.85 ± 0.98 ^Ab^	53.53 ± 1.31 ^Aa^
T2	16.32 ± 0.10 ^Aa^	14.46 ± 0.24 ^Ab^	1.75 ± 0.06 ^Aa^	1.52 ± 0.05 ^Ab^	47.32 ± 1.21 ^Ab^	54.37 ± 1.15 ^Aa^
T3	16.75 ± 0.09 ^Aa^	14.52 ± 0.18 ^Ab^	1.74 ± 0.05 ^Aa^	1.57 ± 0.08 ^Ab^	43.54 ± 2.01 ^Ab^	52.98 ± 1.05 ^Aa^
	Zn content (mg⋅kg^−1^)		Cu content (mg⋅kg^−1^)		Mn content (mg⋅kg^−1^)	
Unextruded	37.54 ± 0.72 ^Aa^	24.53 ± 0.21 ^Ab^	6.02 ± 0.19 ^Ba^	4.73 ± 0.05 ^Bb^	48.67 ± 0.40 ^Ba^	45.32 ± 0.97 ^Ab^
T1	37.98 ± 1.03 ^Aa^	25.06 ± 0.97 ^Ab^	6.97 ± 0.16 ^Aa^	5.63 ± 0.14 ^Ab^	54.36 ± 1.86 ^Aa^	46.86 ± 1.54 ^Ab^
T2	38.73 ± 2.35 ^Aa^	24.75 ± 1.21 ^Ab^	6.85 ± 0.17 ^Aa^	5.57 ± 0.27 ^Ab^	56.75 ± 1.54 ^Aa^	47.37 ± 2.13 ^Ab^
T3	37.06 ± 1.32 ^Aa^	25.57 ± 1.03 ^Ab^	7.02 ± 0.23 ^Aa^	5.60 ± 0.26 ^Ab^	55.37 ± 1.23 ^Aa^	45.96 ± 1.06 ^Ab^

BGW, black-grained wheat; WGW, white-grained wheat; WAI, water absorption index; WSI, water solubility index; T1–T5, five treatment levels, see Table 2; ^a^ Extruder barrel temperature in zones 1–5. The data are represented as the mean ± standard deviation (SD). Values with different capital letters in the same column are significantly different (α = 0.01). Values with different small letters in the same row are significantly different (α = 0.05).

**Table 5:** Effect of the extrusion variables on the total starch content of the wheat extrudates.

Extrusion Variables	Treatments	Total Starch Content (%)	
		BGW	WGW
Barrel temperature (°C) ^a^	Unextruded	57.50 ± 0.69 ^Ab^	69.64 ± 0.54 ^Aa^
	T1	55.43 ± 0.64 ^ABb^	67.52 ± 0.86 ^ABa^
	T2	54.51 ± 0.41 ^BCb^	66.55 ± 0.63 ^BCa^
	T3	52.29 ± 0.80 ^CDb^	64.49 ± 0.61 ^CDa^
	T4	51.30 ± 0.79 ^Db^	63.77 ± 0.76 ^Da^
	T5	50.39 ± 0.70 ^Db^	62.67± 0.42 ^Da^
Feed moisture content (%)	Unextruded	57.50 ± 0.69 ^Ab^	69.64 ± 0.54 ^Aa^
	15.0	55.47 ± 0.53 ^ABb^	67.63 ± 0.73 ^ABa^
	17.5	54.69 ± 0.55 ^BCb^	66.92 ± 0.47 ^BCa^
	20.0	52.76 ± 0.54 ^CDb^	64.93 ± 0.85 ^CDa^
	22.5	52.46 ± 0.79 ^CDb^	64.49 ± 0.51 ^Da^
	25.0	51.86 ± 0.76 ^Db^	63.92 ± 0.73 ^Da^
Feed rate (g·min^−1^)	Unextruded	57.50 ± 0.69 ^Ab^	69.64 ± 0.54 ^Aa^
	20.0	55.60 ± 0.66 ^ABb^	67.76 ± 0.65 ^ABa^
	30.0	54.73 ± 0.60 ^BCb^	66.88 ± 0.76 ^BCa^
	40.0	53.03 ± 0.72 ^CDb^	64.79 ± 0.75 ^CDa^
	50.0	52.41 ± 0.77 ^CDb^	64.30 ± 0.69 ^CDa^
	60.0	51.93 ± 0.76 ^Db^	63.74 ± 0.68 ^Da^

BGW, black-grained wheat; WGW, white-grained wheat; T1–T5, five treatment levels, see Table 2; ^a^ Extruder barrel temperature in zones 1–5. The data are represented as the mean ± standard deviation (SD). Values with different capital letters in the same column are significantly different (α = 0.01). Values with different small letters in the same row are significantly different (α = 0.05).

**Table 6:** Effect of the feed moisture content on the physical properties and nutritional components of the wheat extrudates.

Feed Moisture Content (%)	Specific Volume (m^3^·kg^−1^)		Expansion Ratio (%)		Hardness (N)	
	BGW	WGW	BGW	WGW	BGW	WGW
15.0	1.36 ± 0.08 ^Cb^	2.16 ± 0.06 ^Ca^	0.51 ± 0.06 ^Ca^	0.36 ± 0.02 ^Bb^	6.60 ± 0.42 ^Aa^	6.91 ± 0.30 ^Aa^
17.5	2.56 ± 0.10 ^Bb^	3.02 ± 0.05 ^Ba^	0.78 ± 0.03 ^Ba^	0.68 ± 0.03 ^Ab^	4.53 ± 0.33 ^Ca^	4.43 ± 0.30 ^Ca^
20.0	3.21 ± 0.08 ^Ab^	3.48 ± 0.05 ^Aa^	0.99 ± 0.05 ^Aa^	0.75 ± 0.01 ^Ab^	3.80 ± 0.42 ^Ca^	4.00 ± 0.25 ^Ca^
22.5	2.46 ± 0.07 ^Bb^	2.87 ± 0.04 ^Ba^	0.72 ± 0.06 ^Ba^	0.65 ± 0.04 ^Aa^	5.38 ± 0.36 ^Bb^	4.56 ± 0.45 ^Ca^
25.0	1.45 ± 0.05 ^Cb^	2.22 ± 0.06 ^Ca^	0.54 ± 0.04 ^Ca^	0.42 ± 0.03 ^Ba^	5.47 ± 0.32 ^Bb^	5.15 ± 0.18 ^Ba^
	Fracturability (mm)		WAI (%)		WSI (%)	
15.0	−2.11 ± 0.09 ^Ca^	−1.92 ± 0.09 ^Ca^	5465.00 ± 70.32 ^Cb^	5576.00 ± 50.75 ^Cb^	275.45 ± 18.75 ^Aa^	255.34 ± 19.46 ^Aa^
17.5	−1.04 ± 0.06 ^Aa^	−0.93 ± 0.05 ^Aa^	6056.00 ± 60.34 ^Ba^	6357.00 ± 64.35 ^Ba^	220.16 ± 16.87 ^Ba^	207.54 ± 15.75 ^Ba^
20.0	−1.00 ± 0.08 ^Aa^	−0.85 ± 0.04 ^Aa^	6485.00 ± 57.98 ^Ab^	6786.00 ± 60.85 ^Aa^	235.23 ± 22.34 ^Ba^	186.57 ± 20.71 ^Bb^
22.5	−1.47 ± 0.07 ^Ba^	−1.38 ± 0.06 ^Ba^	6542.00 ± 82.79 ^Ab^	6897.00 ± 71.21 ^Aa^	157.06 ± 15.70 ^Ca^	155.56 ± 24.77 ^Ca^
25.0	−1.95 ± 0.06 ^Ca^	−1.86 ± 0.04 ^Ca^	6638.00 ± 46.18 ^Ab^	7089.00 ± 50.91 ^Aa^	138.65 ± 25.18 ^Ca^	140.62 ± 11.12 ^Ca^
	Protein content (%)		Ash content (%)		Fe content (mg⋅kg^−1^)	
Unextruded	16.53 ± 0.01 ^Aa^	14.24 ± 0.09 ^Ab^	1.71 ± 0.01 ^Aa^	1.53 ± 0.06 ^Ab^	33.56 ± 1.32 ^Bb^	43.76 ± 0.34 ^Ba^
20.0	16.35 ± 0.05 ^Aa^	14.26 ± 0.05 ^Ab^	1.72 ± 0.05 ^Aa^	1.55 ± 0.04 ^Ab^	42.76 ± 1.01 ^Ab^	52.96 ± 1.76 ^Aa^
22.5	16.64 ± 0.06 ^Aa^	14.38 ± 0.07 ^Ab^	1.68 ± 0.08 ^Aa^	1.49 ± 0.04 ^Ab^	43.65 ± 1.06 ^Ab^	53.06 ± 2.14 ^Aa^
25.0	16.28 ± 0.08 ^Aa^	14.50 ± 0.09 ^Ab^	1.75 ± 0.05 ^Aa^	1.51 ± 0.08 ^Ab^	43.97 ± 1.12 ^Ab^	54.13 ± 2.42 ^Aa^
	Zn content (mg⋅kg^−1^)		Cu content (mg⋅kg^−1^)		Mn content (mg⋅kg^−1^)	
Unextruded	37.54 ± 0.72 ^Aa^	24.53 ± 0.21 ^Ab^	6.02 ± 0.19 ^Ba^	4.73 ± 0.05 ^Bb^	48.67 ± 0.40 ^Aa^	45.32 ± 0.97 ^Ab^
20.0	37.86 ± 0.79 ^Aa^	25.43 ± 1.02 ^Ab^	6.85 ± 0.14 ^Aa^	5.57 ± 0.36 ^Ab^	50.13 ± 0.43 ^Aa^	46.32 ± 1.76 ^Ab^
22.5	38.79 ± 1.13 ^Aa^	23.86 ± 0.87 ^Ab^	6.73 ± 0.28 ^Aa^	5.74 ± 0.68 ^Ab^	51.24 ± 0.75 ^Aa^	47.52 ± 1.86 ^Ab^
25.0	39.75 ± 1.65 ^Aa^	25.07 ± 1.21 ^Ab^	6.71 ± 0.12 ^Aa^	5.63 ± 0.32 ^Ab^	49.53 ± 1.04 ^Aa^	45.97 ± 2.01 ^Aa^

BGW, black-grained wheat; WGW, white-grained wheat; WAI, water absorption index; WSI, water solubility index. The data are represented as the mean ± standard deviation (SD). Values with different capital letters in the same column are significantly different (α = 0.01). Values with different small letters in the same row are significantly different (α = 0.05).

**Table 7:** Effect of the feed rate on the physical properties and nutritional components of the wheat extrudates.

Feed Rate (g·min^−1^)	Specific Volume (m^3^·kg^−1^)		Expansion Ratio (%)		Hardness (N)	
	BGW	WGW	BGW	WGW	BGW	WGW
20.0	2.38 ± 0.04 ^Ca^	2.21 ± 0.01 ^Ca^	0.66 ± 0.07 ^Ba^	0.53 ± 0.04 ^Ba^	7.38 ± 0.31 ^Aa^	7.01 ± 0.30 ^Aa^
30.0	2.87 ± 0.04 ^Ba^	2.86 ± 0.06 ^Ba^	0.72 ± 0.04 ^Ba^	0.70 ± 0.03 ^Aa^	4.34 ± 0.30 ^Ca^	4.45 ± 0.43 ^Ca^
40.0	3.26 ± 0.06 ^Ab^	3.48 ± 0.03 ^Aa^	0.97 ± 0.03 ^Aa^	0.72 ± 0.05 ^Ab^	3.83 ± 0.35 ^Ca^	4.06 ± 0.32 ^Ca^
50.0	2.82 ± 0.05 ^Bb^	3.01 ± 0.03 ^Ba^	0.73 ± 0.05 ^Ba^	0.66 ± 0.04 ^Aa^	4.55 ± 0.42 ^Ca^	4.63 ± 0.36 ^Ca^
60.0	2.37 ± 0.03 ^Ca^	2.32 ± 0.05 ^Ca^	0.62 ± 0.05 ^Ba^	0.43 ± 0.03 ^Bb^	5.33 ± 0.20 ^Bb^	6.09 ± 0.24 ^Ba^
	Fracturability (mm)		WAI (%)		WSI (%)	
20.0	−2.18 ± 0.06 ^Cb^	−1.63 ± 0.05 ^Ca^	6335.00 ± 57.98 ^Aa^	6468.00 ± 73.36 ^Aa^	190.32 ± 23.32 ^Ba^	202.18 ± 13.60 ^Ba^
30.0	−1.31 ± 0.08 ^Ba^	−0.96 ± 0.06 ^Aa^	6285.00 ± 37.59 ^Aa^	6407.00 ± 70.71 ^Aa^	214.57 ± 16.31 ^Ba^	216.80 ± 24.89 ^Ba^
40.0	−0.95 ± 0.07 ^Aa^	−0.85 ± 0.03 ^Aa^	6403.00 ± 53.74 ^Ab^	6602.00 ± 78.26 ^Aa^	235.68 ± 16.59 ^Ba^	186.53 ± 25.48 ^Bb^
50.0	−1.45 ± 0.09 ^Ba^	−1.26 ± 0.05 ^Ba^	6123.00 ± 44.34 ^Aa^	6353.00 ± 86.34 ^Aa^	250.54 ± 20.87 ^Ba^	235.43 ± 20.65 ^Ba^
60.0	−2.03 ± 0.14 ^Cb^	−1.68 ± 0.08 ^Ca^	5746.00 ± 57.63 ^Ba^	5976.00 ± 68.55 ^Ba^	312.32 ± 18.65 ^Aa^	315.63 ± 16.54 ^Aa^
	Protein content (%)		Ash content (%)		Fe content (mg⋅kg^−1^)	
Unextruded	16.53 ± 0.01 ^Aa^	14.24 ± 0.09 ^Ab^	1.71 ± 0.01 ^Aa^	1.53 ± 0.06 ^Ab^	33.56 ± 1.32 ^Bb^	43.76 ± 0.34 ^Ba^
20.0	16.82 ± 0.11 ^Aa^	14.34 ± 0.02 ^Ab^	1.75 ± 0.02 ^Aa^	1.55 ± 0.02 ^Ab^	41.36 ± 1.62 ^Ab^	54.17 ± 0.84 ^Aa^
30.0	16.63 ± 0.10 ^Aa^	14.57 ± 0.07 ^Ab^	1.74 ± 0.02 ^Aa^	1.52 ± 0.02 ^Ab^	42.43 ± 1.43 ^Ab^	55.19 ± 1.30 ^Aa^
40.0	16.45 ± 0.08 ^Aa^	14.62 ± 0.08 ^Ab^	1.73 ± 0.03 ^Aa^	1.56 ± 0.02 ^Ab^	40.78 ± 1.25 ^Ab^	53.24 ± 2.45 ^Aa^
	Zn content (mg⋅kg^−1^)		Cu content (mg⋅kg^−1^)		Mn content (mg⋅kg^−1^)	
Unextruded	37.54 ± 0.72 ^Aa^	24.53 ± 0.21 ^Ab^	6.02 ± 0.19 ^Ba^	4.73 ± 0.05 ^Bb^	48.67 ± 0.40 ^Aa^	45.32 ± 0.97 ^Ab^
20.0	37.24 ± 2.11 ^Aa^	24.16 ± 0.33 ^Ab^	6.86 ± 0.10 ^Aa^	5.03 ± 0.25 ^Ab^	49.84 ± 0.41 ^Aa^	44.28 ± 0.55 ^Ab^
30.0	37.96 ± 0.34 ^Aa^	25.36 ± 2.05 ^Ab^	7.01 ± 0.24 ^Aa^	5.12 ± 0.21 ^Ab^	50.98 ± 1.06 ^Aa^	45.73 ± 0.61 ^Ab^
40.0	38.32 ± 0.54 ^Aa^	26.22 ± 2.63 ^Ab^	6.95 ± 0.11 ^Aa^	5.87 ± 0.24 ^Ab^	52.55 ± 2.12 ^Aa^	46.27 ± 1.64 ^Ab^

BGW, black-grained wheat; WGW, white-grained wheat; WAI, water absorption index; WSI, water solubility index. The data are represented as the mean ± Scheme 0. Values with different capital letters in the same column are significantly different (α = 0.01). Values with different small letters in the same row are significantly different (α = 0.05).

**Table 8:** ANOVA of the different models of the responses of the whole BGW extrudates.

Source	Sum of Squares					
	Specific Volume (m^3^·kg^−1^)	Expansion Ratio (%)	Hardness (N)	Fracturability (mm)	WAI (%)	WSI (%)
Model	6.95 ^****^	0.39 ^****^	36.61 ^****^	1.95 ^****^	1.53*10^6 **^	2.06*10^4 ***^
Barrel temperature (*X_1_*)	4.61 ^****^	0.23 ^****^	27.71 ^****^	0.12 ^***^	3.61*10^5 **^	644.94 ^*^
Feed moisture content (*X_2_*)	0.35 ^****^	0.05 ^**^	0.33	0.34 ^****^	2.26*10^5 **^	2.46*10^3 **^
Feed rate (*X_3_*)	0.03 ^**^	6.61*10^−3 *^	1.64 ^**^	0.17 ^***^	6.60*10^3^	106.43
Barrel temperature × Feed moisture content (*X_1_X_2_*)	0.23*10^−3^	0.90*10^−3^	0.42^3 *^	0.40*10^−3^	4.29*10^4^	5.95
Barrel temperature × Feed rate (*X_1_X_3_*)	2.50*10^−3^	0.23*10^−3^	0.068	2.03*10^−3^	1.44*10^3^	133.52
Feed moisture content × Feed rate (*X_2_X_3_*)	0.23*10^−3^	0.40*10^−3^	0.036	0.40*10^−3^	285.72	0.10
Barrel temperature × barrel temperature (*X_1_*^2^)	0.77 ^****^	0.03 ^***^	2.84 ^***^	0.25 ^****^	7.05*10^5 ***^	5.07*10^3 ***^
Feed moisture content × feed moisture content (*X_2_*^2^)	0.53 ^****^	0.03 ^***^	0.61 ^*^	0.12 ^***^	1.17*10^5 *^	7.01*10^3 ****^
Feed rate × feed rate (*X_3_*^2^)	0.74 ^****^	0.04 ^***^	2.36 ^***^	0.83 ^****^	1.63*10^4^	3.35*10^3 ***^
Lack of fit	4.88*10^−3^	5.73*10^−3^	0.36	0.02	9.23*10^4^	370.34
Pure error	2.28*10^−3^	1.48*10^−3^	0.76	6.12*10^−3^	3.30*10^4^	265.37
CV (%)	1.06	3.65	5.23	4.43	2.20	5.43
*R* ^2^	0.98	0.96	0.97	0.97	0.83	0.93

BGW, black-grained wheat; WAI, water absorption index; WSI, water solubility index; * *p* < 0.05; ** *p* < 0.01; *** *p* < 0.001; ****p < 0.0001.

## Data Availability

Data is contained within the article.

## References

[1] Chandra A.K., Kumar A., Bharati A., Joshi R., Agrawal A., Kumar S. (2020). Microbial-Assisted and Genomic-Assisted Breeding: A Two Way Approach for the Improvement of Nutritional Quality Traits in Agricultural Crops. 3 Biotech.

[2] Statista-The Statistic Portal 2020. www.statista.com/statistics/267268/production-of-wheat-worldwide-since-1990/.

[3] Liu H., Wang Z.H., Li F.C., Li K.Y., Yang N., Yang Y., Huang D.L., Liang D.L., Zhao H.B., Mao H. (2014). Grain Iron and Zinc Concentrations of Wheat and Their Relationships to Yield in Major Wheat Production Areas in China. Field Crops Res..

[4] Gomez-Coronado F., Almeida A.S., Santamaria O., Cakmak I., Poblaciones M.J. (2019). Potential of Advanced Breeding Lines of Bread Making Wheat to Accumulate Grain Minerals (Ca, Fe, Mg and Zn) and Low Phytates under Mediterranean Conditions. J. Agron. Crop Sci..

[5] Maltzahn L.E., Zenker S.G., Lopes J.L., Pereira R.M., Verdi C.A., Rother V., Busanello C., Viana V.E., Batista B.L., de Oliveira A.C. (2020). Brazilian Genetic Diversity for Desirable and Undesirable Elements in the Wheat Grain. Biol. Trace Elem. Res..

[6] International Food Policy Research Institute (2017). Global Nutrition Report.

[7] International Food Policy Research Institute (2020). Global Nutrition Report in the Context of COVID-19.

[8] Guo Z.F., Xu P., Zhang Z.B., Wang D.W., Jin M., Teng A.P. (2011). Segregation Ratios of Colored Grains in Crossed Wheat. Aust. J. Crop Sci..

[9] Tian S.Q., Chen Z.C., Wei Y.C. (2018). Measurement of Colour-Grained Wheat Nutrient Compounds and the Application of Combination Technology in Dough. J. Cereal Sci..

[10] Ficco D.B., de Simone V., Colecchia S.A., Pecorella I., Platani C., Nigro F., Finocchiaro F., Papa R., de Vita P. (2014). Genetic Variability in Anthocyanin Composition and Nutritional Properties of Blue, Purple, and Red Bread (Triticum aestivum L.) and Durum (Triticum turgidum L. ssp. turgidum convar. Durum) Wheats. J. Agric. Food Chem..

[11] Ma D.Y., Li Y.G., Zhang J., Wang C.Y., Qin H.X., Ding H.N., Xie Y.X., Guo T.C. (2016). Accumulation of Phenolic Compounds and Expression Profiles of Phenolic Acid Biosynthesis-Related Genes in Developing Grains of White, Purple, and Red Wheat. Front. Plant Sci..

[12] Phuong L.M., Lachman J., Kotikova Z., Orsak M., Michlova T., Martinek P. (2017). Selenium in Colour-Grained Winter Wheat and Spring Tritordeum. Plant Soil Environ..

[13] Francavilla A., Joye I.J. (2020). Anthocyanins in Whole Grain Cereals and Their Potential Effect on Health. Nutrients.

[14] Li L.H., Yang G.P., Ren M.J., Wang Z.N., Peng Y.S., Xu R.H. (2020). Co-Regulation of Auxin and Cytokinin in Anthocyanin Accumulation during Natural Development of Purple Wheat Grains. J. Plant Growth Regul..

[15] Morgounov A., Karaduman Y., Akin B., Aydogan S., Baenziger P.S., Bhatta M., Chudinov V., Dreisigacker S., Govindan V., Guler S. (2020). Yield and Quality in Purple-Grained Wheat Isogenic Lines. Agronomy.

[16] Paznocht L., Buresova B., Kotikova Z., Martinek P. (2020). Carotenoid Content of Extruded and Puffed Products Made of Colored-Grain Wheats. Food Chem..

[17] Paznocht L., Kotikova Z., Buresova B., Lachman J., Martinek P. (2020). Phenolic Acids in Kernels of Different Coloured-Grain Wheat Genotypes. Plant Soil Environ..

[18] Chen X., Li X., Zhu X., Wang G., Zhuang K., Wang Y., Ding W. (2020). Optimization of Extrusion and Ultrasound-Assisted Extraction of Phenolic Compounds from Jizi439 Black Wheat Bran. Processes.

[19] Saini P., Kumar N., Kumar S., Mwaurah P.W., Panghal A., Attkan A.K., Singh V.K., Garg M.K., Singh V. (2020). Bioactive Compounds, Nutritional Benefits and Food Applications of Colored Wheat: A Comprehensive Review. Crit. Rev. Food Sci..

[20] Li W.D., Beta T., Sun S.C., Corke H. (2006). Protein Characteristics of Chinese Black-Grained Wheat. Food Chem..

[21] Li W.D., Shan F., Sun S.C., Corke H., Beta T. (2005). Free Radical Scavenging Properties and Phenolic Content of Chinese Black-Grained Wheat. J. Agric. Food Chem..

[22] Li W.D., Beta T., Preedy V.R., Watson R.R., Patel V.B. (2011). Flour and Bread from Black-, Purple-, and Blue-Colored Wheats. Flour and Breads and Their Fortification in Health and Disease Prevention.

[23] Ma D.Y., Sun D.X., Zuo Y., Wang C.Y., Zhu Y.J., Guo T.C. (2014). Diversity of Antioxidant Content and Its Relationship to Grain Color and Morphological Characteristics in Winter Wheat Grains. J. Integr. Agr..

[24] Chen Z. (2012). Analysis on the Distribution of Main Physicochemical Parameters of Color Wheat and Study on Its Layering Milling Technology. Agric. Sci. Technol..

[25] Saloni S., Venkatesh C., Aman K., Rohit K., Pragyanshu K., Kanthi K.K., Mahendra B., Monika G. (2018). Anthocyanin Bio-Fortified Colored Wheat: Nutritional and Functional Characterization. PLoS ONE.

[26] Liu Y.X., Liu M.M., Zhang Z.M. (2020). Research Progress in Breeding and Nutritional and Processing Quality of Black-Grain Wheat Varsities (Triticom aestivom L.). J. Triticeae Crops.

[27] Liu Y., Qiu J., Yue Y., Li K., Ren G. (2018). Dietary Black-Grained Wheat Intake Improves Glycemic Control and Inflammatory Profile in Patients with Type 2 Diabetes: A Randomized Controlled Trial. Ther. Clin. Risk Manag..

[28] Okarter N., Liu R.H. (2010). Health Benefits of Whole Grain Phytochemicals. Crit. Rev. Food Sci. Nutr..

[29] Ficco D.B.M., Beleggia R., Pecorella I., Giovanniello V., Frenda A.S., Vita P.D. (2020). Relationship between Seed Morphological Traits and Ash and Mineral Distribution along the Kernel Using Debranning in Durum Wheats from Different Geographic Sites. Foods.

[30] Gómez M., Gutkoski L.C., Bravo-Núñez Á. (2020). Understanding Whole-Wheat Flour and Its Effect in Breads: A Review. Compr. Rev. Food Sci. Food Saf..

[31] Cheftel J.C. (1986). Nutritional Effects of Extrusion-Cooking. Food Chem..

[32] Tran Q.D., Hendriks W.H., van der Poel A.F.B. (2008). Effects of Extrusion Processing on Nutrients in Dry Pet Food. J. Sci. Food Agric..

[33] Altan A., Mccarthy K.L., Maskan M. (2008). Extrusion Cooking of Barley Flour and Process Parameter Optimization by Using Response Surface Methodology. J. Sci. Food Agric..

[34] Arora B., Yoon A., Sriram M., Singha P., Rizvi S.S.H. (2020). Reactive Extrusion: A Review of the Physicochemical Changes in Food Systems. Innov. Food Sci. Emerg..

[35] Thymi S., Krokida M.K., Pappa A., Maroulis Z.B. (2005). Structural Properties of Extruded Corn Starch. J. Food. Eng..

[36] Drago S.R., Velasco-GonzÁlez O.H., Torres R.L., González R.J., Valencia M.E. (2007). Effect of the Extrusion on Functional Properties and Mineral Dialyzability from Phaseolus Vulgaris Bean Flour. Plant Foods Hum. Nutr..

[37] Jing Y., Chi Y. (2013). Effects of Twin-Screw Extrusion on Soluble Dietary Fiber and Physicochemical Properties of Soybean Residue. Food Chem..

[38] Gandhi N., Singh B., Singh P., Sharma S. (2020). Functional, Rheological, Morphological, and Micro-Structural Properties of Extrusion-Processed Corn and Potato Starches. Starch Stärke.

[39] Wang Q.F., Li L.M., Zheng X.L., Xiong X.Q. (2020). Effect of Extrusion Feeding Moisture on Dough, Nutritional, and Texture Properties of Noodles Fortified with Extruded Buckwheat Flour. J. Food Process. Pres..

[40] Singh S., Gamlath S., Wakeling L. (2007). Nutritional Aspects of Food Extrusion: A Review. Int. J. Food Sci. Technol..

[41] Anderson A.K., Ng P.K.W. (2000). Changes in Disulfide and Sulfhydryl Contents and Electrophoretic Patterns of Extruded Wheat Flour Proteins. Cereal Chem..

[42] Myat L., Ryu G.H. (2014). Characteristics of Destarched Corn Fiber Extrudates for Ethanol Production. J. Cereal Sci..

[43] Imran M., Anjum F.M., Butt M.S., Sheikh M.A. (2014). Influence of Extrusion Processing on Tannin Reduction and Oil Loss in Flaxseed (Linum usitatissimum L.) Meal. J. Food Process. Pres..

[44] Kristiawan M., Micard V., Maladira P., Alchamieh C., Maigret J.-E., Réguerre A.-L., Emin M.A., Della Valle G. (2018). Multi-Scale Structural Changes of Starch and Proteins during Pea Flour Extrusion. Food Res. Int..

[45] Brennan M.A., Derbyshire E., Tiwari B.K., Brennan C.S. (2013). Ready-to-Eat Snack Products: The Role of Extrusion Technology in Developing Consumer Acceptable and Nutritious Snacks. Int. J. Food Sci. Technol..

[46] Tabibloghmany F.S., Mazaheri Tehrani M., Koocheki A. (2020). Optimization of the Extrusion Process through Response Surface Methodology for Improvement in Functional and Nutritional Properties of Soybean Hull. J. Food Sci. Technol..

[47] Ding Q.B., Ainsworth P., Plunkett A., Tucker G., Marson H. (2006). The Effect of Extrusion Conditions on the Functional and Physical Properties of Wheat-Based Expanded Snacks. J. Food Eng..

[48] Balasubramanian S., Borah A., Singh K.K., Patil R.T. (2012). Effect of Selected Dehulled Legume Incorporation on Functional and Nutritional Properties of Protein Enriched Sorghum and Wheat Extrudates. J. Food Sci. Technol..

[49] Singh R.K.R., Majumdar R.K., Venkateshwarlu G. (2014). Effect of Process Conditions on Physico-Chemical and Sensory Properties of Fish-Cereal-Based Extruded Snack-Like Products. J. Food Process. Pres..

[50] Moraru C.I., Kokini J.L. (2003). Nucleation and Expansion During Extrusion and Microwave Heating of Cereal Foods. Compr. Rev. Food Sci. Food Saf..

[51] Anderson A.K., Ng P.K.W. (2003). Physical and Microstructural Properties of Wheat Flour Extrudates as Affected by Vital Gluten Addition and Process Conditions. Food Sci. Biotechnol..

[52] Kirby A.R., Ollett A.L., Parker R., Smith A.C. (1988). An Experimental Study of Screw Configuration Effects in the Twin-Screw Extrusion-Cooking of Maize Grits. J. Food Eng..

[53] Sarifudin A., Assiry A.M. (2014). Some Physicochemical Properties of Dextrin Produced by Extrusion Process. J. Saudi Soc. Agric. Sci..

[54] Roye C., Henrion M., Chanvrier H., de Roeck K., de Bondt Y., Liberloo I., King R., Courtin C.M. (2020). Extrusion-Cooking Modifies Physicochemical and Nutrition-Related Properties of Wheat Bran. Foods.

[55] Vaz L.C.M.A., Arêas J.A.G. (2010). Recovery and Upgrading Bovine Rumen Protein by Extrusion: Effect of Lipid Content on Protein Disulphide Cross-Linking, Solubility and Molecular Weight. Meat Sci..

[56] Mosibo O.K., Ferrentino G., Alam M.R., Morozova K., Scampicchio M. (2020). Extrusion Cooking of Protein-Based Products: Potentials and Challenges. Crit. Rev. Food Sci. Nutr..

[57] Angelis D.D., Kaleda A., Pasqualone A., Vaikma H., Tamm M., Tammik M.L., Squeo G., Summo C. (2020). Physicochemical and Sensorial Evaluation of Meat Analogues Produced from Dry-Fractionated Pea and Oat Proteins. Foods.

[58] Guldiken B., Yovchev A., Nosworthy M.G., Stone A.K., House J.D., Hood-Niefer S., Nickerson M.T. (2020). Effect of Extrusion Conditions on the Physical Properties of Desi Chickpea-Barley Extrudates and Quality Attributes of Their Resulting Flours. J. Texture Stud..

[59] Fang Y., Zhang B., Wei Y., Li S. (2013). Effects of Specific Mechanical Energy on Soy Protein Aggregation during Extrusion Process Studied by Size Exclusion Chromatography Coupled with Multi-Angle Laser Light Scattering. J. Food Eng..

[60] Koch L., Hummel L., Schuchmann H.P., Emin M.A. (2018). Improving the Emulsifying Properties of Whey Protein Isolate-Citrus Pectin Blends by a Novel Reactive Extrusion Approach. J. Food Eng..

[61] Pasqualone A., Costantini M., Coldea T.E., Summo C. (2020). Use of Legumes in Extrusion Cooking: A Review. Foods.

[62] Camire M.E., Shahidi F., Ho C.T., van Chuyen N. (1998). Chemical Changes during Extrusion Cooking. Process-Induced Chemical Changes in Food. Advances in Experimental Medicine and Biology.

[63] Chen F.L., Chen X.Y., Wang X.C., Yin J., Zhao X.Y. (2013). Relationship between Wheat Quality and Physicochemical Properties of Extruded Expansion Product. Sci. Agric. Sin..

[64] Zahari I., Ferawati F., Helstad A., Ahlstrom C., Ostbring K., Rayner M., Purhagen J.K. (2020). Development of High-Moisture Meat Analogues with Hemp and Soy Protein Using Extrusion Cooking. Foods.

[65] Ramzan Y., Hafeez M.B., Khan S., Nadeem M., Batool S., Ahmad J. (2020). Biofortification with Zinc and Iron Improves the Grain Quality and Yield of Wheat Crop. Int. J. Plant Prod..

[66] van den Einde R.M., van der Veen M.E., Bosman H., van der Goot A.J., Boom R.M. (2005). Modeling Macromolecular Degradation of Corn Starch in a Twin Screw Extruder. J. Food Eng..

[67] Bu C.W. (2011). Operating Parameters’ Influence on the Specific Volume of Extrusion Puffed Food. Appl. Mech. Mater..

[68] Nyombaire G., Siddiq M., Dolan K.D. (2011). Physico-Chemical and Sensory Quality of Extruded Light Red Kidney Bean (Phaseolus vulgaris L.) Porridge. LWT Food Sci. Technol..

[69] Badrie N., Mellowes W.A. (2010). Texture and Microstructure of Cassava (Manihot Esculenta Crantz) Flour Extrudate. J. Food Sci..

[70] Kumar A., Samuel D.V.K., Jha S.K., Sinha J.P. (2015). Twin Screw Extrusion of Sorghum and Soya Blends: A Response Surface Analysis. J. Agric. Sci. Technol..

[71] Myers R., Montgomery D.C. (2002). Response Surface Methodology: Process and Products Optimization Using Designed Experiments.

